# Intensive care unit-to-unit capacity transfers are associated with increased mortality: no hasty conclusions in the event of a crisis

**DOI:** 10.1186/s13613-022-01031-7

**Published:** 2022-07-02

**Authors:** Benoit Painvin, Stephan Ehrmann, Arnaud W. Thille, Jean-Marc Tadié

**Affiliations:** 1 Service des Maladies Infectieuses et de Réanimation Médicale, Centre Hospitalier Universitaire de Rennes, Hôpital Pontchaillou, 2 rue Henri le Guilloux, 35033 Rennes, France; 2 Service de Médecine Intensive Et Réanimation, CRICS- Triggersep Research Network, Centre Hospitalier Régional Universitaire de Tours, CIC INSERM 1415Hôpital Bretonneau 2, boulevard Tonnellé, 27044 Tours, France; 3 Centre d’étude des pathologies respiratoires, INSERM U1100, Université de Tours, Tours, France; 4 Service de Médecine Intensive Et Réanimation, Centre Hospitalier Universitaire de Poitiers, 2 rue de la Milétrie, 90577 86000 Poitiers, France; 5 Faculté de Médecine, Université de Rennes 1, Unité INSERM CIC 1414, IFR 140, Rennes, France

Dear Editor,

We read with great interest the study of Parenmark et al., a large retrospective study, including 15,588 ICU-to-ICU interhospital transfers in Sweden over a 2-year period [1].

The authors describe three types of interhospital transfers: clinical transfer (need for specialised care not available in the admitting hospital), capacity transfer (making room for patients with more urgent need for intensive care when all ICU beds are occupied) and repatriation (return to the home ICU following initial treatment at another unit) the last one being labelled as reference. Their main result indicates an increase mortality within 30 days following discharge from the referring ICU in the subgroups of clinical and capacity transfers, with adjusted odds ratio of 1.17 (95% CI 1.02–1.36) and 1.25 (95% CI 1.06–1.49), respectively.

As the authors notice, the main result is somewhat surprising as higher mortality has not been reported in recent literature [2, 3]. Reasons could be explained as follow: first, the authors specify that 20% of capacity transfers occurred at night, involving severe critically ill patients with acute lung injury, sepsis, and cardiogenic shock. In light of these results, one could wonder whether it is safe or not for the patient to undergo a night ICU-to-ICU transfer compared to withholding the interhospital transport for a few hours until the sun rises, since night-shift patient’s discharge has been associated with a higher mortality [4].

Second, the authors pointed out that Sweden has a low number of ICU bed which could play a role in the higher mortality rate found following interhospital capacity and clinical transfers [5].

Solutions to tackle this higher mortality related to interhospital transfer would be to build up local resources for critical care: increasing ICU beds, recruiting ICU highly trained staff and Intensivist doctors to avoid transfers of critically ill patients at nights with severe unstable pathologies (especially during wintertime when respiratory sepsis and acute respiratory distress occur more frequently [6]).

Furthermore, the authors’ message must be balanced when facing crisis, such as the COVID-19 pandemic. Assuming that interhospital transfers are unsafe and choosing a strategy of implementation of new ICU beds to face surge of critically ill patients could lead to a higher mortality [7]. During the first months of the COVID-19 crisis, countries planned and organized large-scale interhospital transfers either for clinical or capacity reasons and demonstrated that transferred patients did not have a higher mortality rate [2, 3, 8]. However, we agree with the authors and acknowledge that “understanding the impact of ICU-to-ICU transfer on patient outcome is complex and must consider a couple of important aspects” such as identifying appropriate control patients.

Finally, as mentioned by the authors, robust prospective studies including before departure, ongoing transport and arrival data are needed to determine the timing of the transfer, the safest medical condition allowing for transfer, and whether transport impacts ICU mortality.

## Data Availability

Not applicable.

## Abbreviations

ICU: Intensive care unit
